# A machine learning model for 90-day mortality prediction in hepatitis B virus-related acute-on-chronic liver failure: the pivotal role of CALLY index

**DOI:** 10.3389/fmed.2026.1814799

**Published:** 2026-07-03

**Authors:** Yijun Zhang, Chunyan Li, Shaohui Su, Jilin Huang, Siyu Fu, Yong Zhang, Shanhong Tang

**Affiliations:** 1 Department of Gastroenterology, The General Hospital of Western Theater Command, Chengdu, Sichuan, China; 2 Department of Gastroenterology, Chengdu Sixth People's Hospital, Chengdu, Sichuan, China; 3 School of Medical and Life Sciences, Chengdu University of Traditional Chinese Medicine, Chengdu, Sichuan, China

**Keywords:** CALLY index, HBV-ACLF, immune-nutritional status, lightgbm, machine learning, shap, systemic inflammation

## Abstract

**Background:**

Hepatitis B virus-related acute-on-chronic liver failure (HBV-ACLF) is a life-threatening syndrome, the condition can deteriorate rapidly, and the 90-day mortality rate is high. Due to the rapid changes in the clinical course, early and accurate risk stratification is crucial for timely decision-making and resource allocation in the ICU. This study has developed and verified a machine learning framework that integrates the C-reactive protein-albumin-lymphocyte (CALLY) index to predict the 90-day mortality rate of HBV-ACLF patients.

**Methods:**

We conducted a retrospective single-center study on 471 patients with HBV-ACLF who met the 2018 Chinese liver failure diagnosis criteria and were hospitalized in the Western Theater General Hospital from 2015 to 2023. We randomly divide the constructed data set into training set (*n* = 329) and internal verification set (*n* = 142) according to the ratio of 7:3. In addition, we performed temporal external validation using a later cohort of 46 patients with HBV-ACLF admitted between 2024 and 2025. Six machine-learning algorithms including logistic regression, support vector machine, K-nearest neighbors, Extra Trees, XGBoost, and LightGBM, were trained to develop predictive models. SHAP was applied to assess feature importance and model interpretability.

**Results:**

Among the evaluated algorithms, the LightGBM model showed the strongest overall discriminative performance, with AUCs of 0.940 (95% CI, 0.916–0.964) in the training set, 0.825 (95% CI, 0.757–0.894) in the internal validation set, and 0.804 (95% CI, 0.669–0.939) in the external validation set. SHAP analysis identified international normalized ratio (INR) as the strongest predictor of mortality, followed by the CALLY index, log-transformed total bilirubin (TBIL), age, and creatinine. The CALLY-based model significantly improved risk stratification compared with the Model for End-Stage Liver Disease (MELD) score, as demonstrated by a continuous net reclassification index of 0.6223 and an integrated discrimination improvement of 0.0937. To facilitate clinical implementation, a user-friendly online tool was created to predict mortality rates.

**Conclusions:**

Our machine-learning framework integrating the CALLY index provides a high-precision and transparent decision-support tool for predicting 90-day mortality with HBV-ACLF patients. By quantifying the immune–nutritional–inflammatory axis, this approach may facilitate earlier risk stratification and personalized interventions, potentially improving survival in high-risk patients.

## 1 Introduction

Hepatitis B virus-related acute-on-chronic liver failure (HBV-ACLF) is a life-threatening syndrome of rapid liver deterioration with very high short-term mortality (1, 2). Patients often progress quickly to multiple organ failure, so intensive support including ICU care and liver replacement therapies must be mobilized immediately (3). Because the window for interventions (such as transplantation) is narrow, early risk stratification is critical (4). In critical care, identifying high-risk patients at admission allows clinicians to allocate scarce ICU resources and organ support most effectively.

Existing prognostic scores (e.g. MELD, Child–Pugh) often miss the complex inflammatory and nutritional dynamics of ACLF (5). The CRP-albumin-lymphocyte (CALLY) index is a novel marker that reflects a patient's immune–nutritional status and has shown promise in liver disease (6, 7). In this study, the CALLY Index is calculated as [(Albumin, g/L × Lymphocyte, 10^9^ cells/L) × 10 / CRP, mg/L]. We hypothesized that integrating the CALLY index into a machine-learning framework could improve early warning of deterioration. Recent work shows that machine-learning (ML) models can capture non-linear patterns in ICU patients and thus help predict outcomes that traditional models miss (8–10).

In this study, we analyzed a cohort of HBV-ACLF patients with baseline labs, demographics and imaging, and we evaluated advanced ML classifiers (e.g., LightGBM) to predict 90-day mortality. We used SHAP (Shapley Additive Explanations) to interpret the model, ensuring transparency in how features like CALLY influence risk. Our goal is a precise, interpretable prediction tool to guide ICU triage and personalized organ-support decisions in HBV-ACLF.

## Methods

### Study design and setting

We conducted a retrospective, single-center cohort study at the General Hospital of Western Theater Command. Patients with hepatitis B virus-related acute-on-chronic liver failure (HBV-ACLF) were identified from the electronic medical record system using a keyword search (“hepatitis B virus” and “ACLF”) supplemented by ICD-10 codes. The derivation cohort included admissions between January 2015 and April 2023. A temporally distinct cohort admitted between January 2024 and December 2025 served as the external validation set (temporal validation).

### Participants

HBV-ACLF was diagnosed according to the 2018 Chinese criteria for liver failure. Eligible patients were 18–80 years of age and had complete 90-day follow-up for ascertainment of the primary endpoint. Patients with non-HBV viral hepatitis, other chronic liver diseases, malignancy (including suspected or confirmed hepatocellular carcinoma), pregnancy/lactation, prior liver transplantation, long-term corticosteroid exposure before ACLF onset, or severely incomplete clinical datasets were excluded. Each hospitalization was assigned a unique identifier, and only the first eligible admission was analyzed to ensure data independence.

### Outcome and candidate predictors

The primary endpoint was 90-day all-cause mortality after HBV-ACLF diagnosis. Candidate predictors were selected a priori based on clinical relevance and published literature, and included demographics (age group, sex), laboratory parameters [total bilirubin, international normalized ratio (INR), serum creatinine, alanine aminotransferase], and quantitative ultrasound-derived features. Laboratory variables were extracted from the earliest available results within 24–48 h of admission. The CALLY index was calculated as: [albumin (g/L) × lymphocyte count (10^9^ cells/L) × 10] / CRP [mg/L]. Total bilirubin was log-transformed (log-TBIL) when used in regression-based modeling.

### Data preprocessing and missing data

Outliers in key continuous variables were screened using the interquartile range (IQR) rule and were handled according to predefined rules (removal if biologically implausible or winsorization if plausibly extreme). Missing data were addressed using multiple imputation by chained equations (MICE) assuming missing at random. Five imputed datasets were generated using predictive mean matching for continuous variables and logistic/polytomous regression for categorical variables, and estimates were pooled using Rubin's rules. Convergence was assessed using trace plots.

### Cohort division, model development and interpretability

In order to carry out internal verification, the derivation cohort is randomly divided into training set (70%) and internal verification set (30%). First, use single-variate analysis to screen candidate predictors (*P* < 0.05). In order to reduce multiple collinearity and improve the conciseness of the model, LASSO regression with 10-fold cross-validation was then applied, and the predictor of non-zero coefficient under the selected penalty term was retained. The predictive factors retained by screening and LASSO were used to fit the multivariate logistic regression model; in view of the clinical significance of serum creatinine, serum creatinine was retained in the final model. A nomogram was constructed based on the CALLY index, log-TBIL, INR, age group and creatinine.

At the same time, using these five predictors, models based on six machine learning (logistic regression, SVM, KNN, Extra Trees, XGBoost and LightGBM) were built. Hyperparameters were optimized using 10-fold cross-validation within the training set. To ensure model generalizability and prevent overfitting, conservative tuning ranges were predefined; the comprehensive search spaces and final optimal configurations for each algorithm are detailed in Supplementary Table 1. Use SHAP to evaluate the interpretability of the model of the best performance classifier to quantify the contribution of global and individual characteristics.

### Model performance, comparison and clinical practicality

Use AUROC to quantify the distinction, and use the bootstrap resampling method (1,000 iterations) to estimate the 95% confidence interval. Use the bootstrap calibration curve that has been corrected by deviation to evaluate the calibration. Use decision-making curve analysis to evaluate clinical practicality. Use NRI and IDI to quantify the incremental predicted value added to CALLY to the MELD score. Use the DeLong test to make a statistical comparison of AUROC. All models use preset model parameters for further evaluation in the external time verification set.

### Statistical analysis

All analyses are carried out in R language (version 4.2.0), and bilateral *P* < 0.05 is considered statistically significant. After the regularity test, the continuous variables are compared by the Student's *t*-test or the Mann-Whitney *U*-test according to the situation; the classified variables are compared by the chi-square test or the Fisher's exact test. The Spearman correlation coefficient is used to evaluate the monotonous correlation between predictors. The survival analysis adopts Kaplan-Meier survival curve and logarithmic rank test. Multiple collinearity is evaluated by the variance inflation factor (VIF), and VIF < 5 is considered acceptable.

## Results

### Baseline characteristics of patients

**Figure 1:**
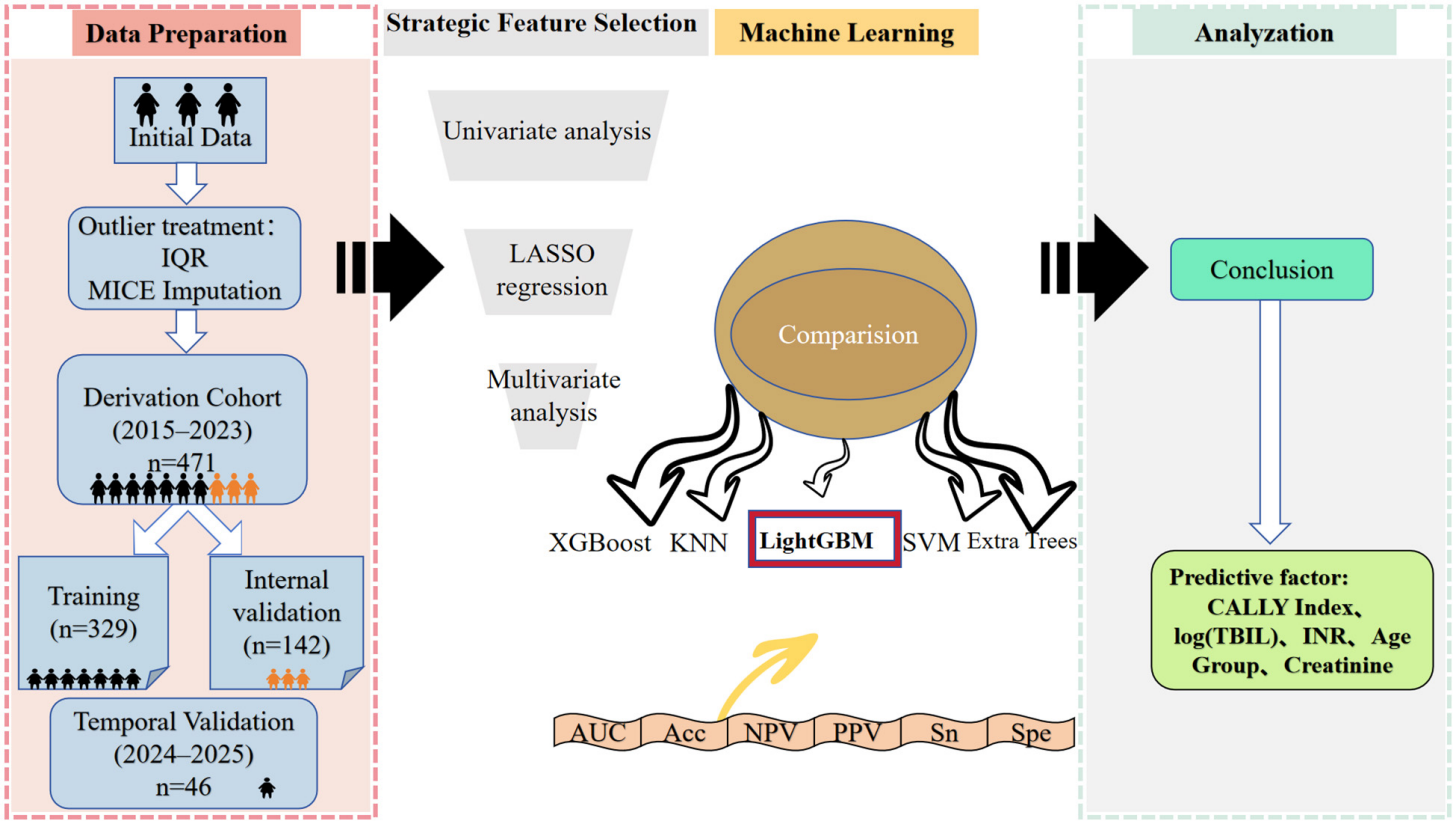
Graphical Abstract.

The research process and cohort division are shown in Figure 1. After strict data cleaning and using IQR method to deal with abnormal values, a total of 471 HBV-ACLF patients were included. There were no significant differences between the two cohorts regarding age, sex, and key clinical severity indicators, including MELD score and ACLF grading (*P* > 0.05), confirming the baseline balance and the representativeness of the randomized split (Table 1).

**Table 1:** Baseline characteristics of HBV-ACLF in the training and internal validation sets.

Variable	Training set (*N* = 329)	Validation set (*N* = 142)	*P*-value
Age (years)	51.00 (44.00–59.00)	50.00 (44.00–61.00)	0.812
TBIL (μmol/L)	296.40 (208.50–404.79)	311.00 (190.88–431.87)	0.578
INR	1.82 (1.59–2.15)	1.79 (1.54–2.18)	0.736
Creatinine (μmol/L)	76.00 (65.00–92.00)	74.50 (63.25–91.00)	0.507
MELD score	22.55 (19.73–25.88)	22.35 (19.05–26.21)	0.773
Gender			0.953
Male	271 (82.4%)	118 (83.1%)	
Female	58 (17.6%)	24 (16.9%)	
ACLF type			0.594
Type A	106 (32.2%)	49 (34.5%)	
Type B	27 (8.2%)	8 (5.6%)	
Type C	159 (48.3%)	68 (47.9%)	
90-day outcome			1
Survival	195 (59.3%)	84 (59.2%)	
Death	134 (40.7%)	58 (40.8%)	
CALLY index	21.69 (12.70–36.89)	23.52 (14.33–42.53)	0.211

**Figure 2:**
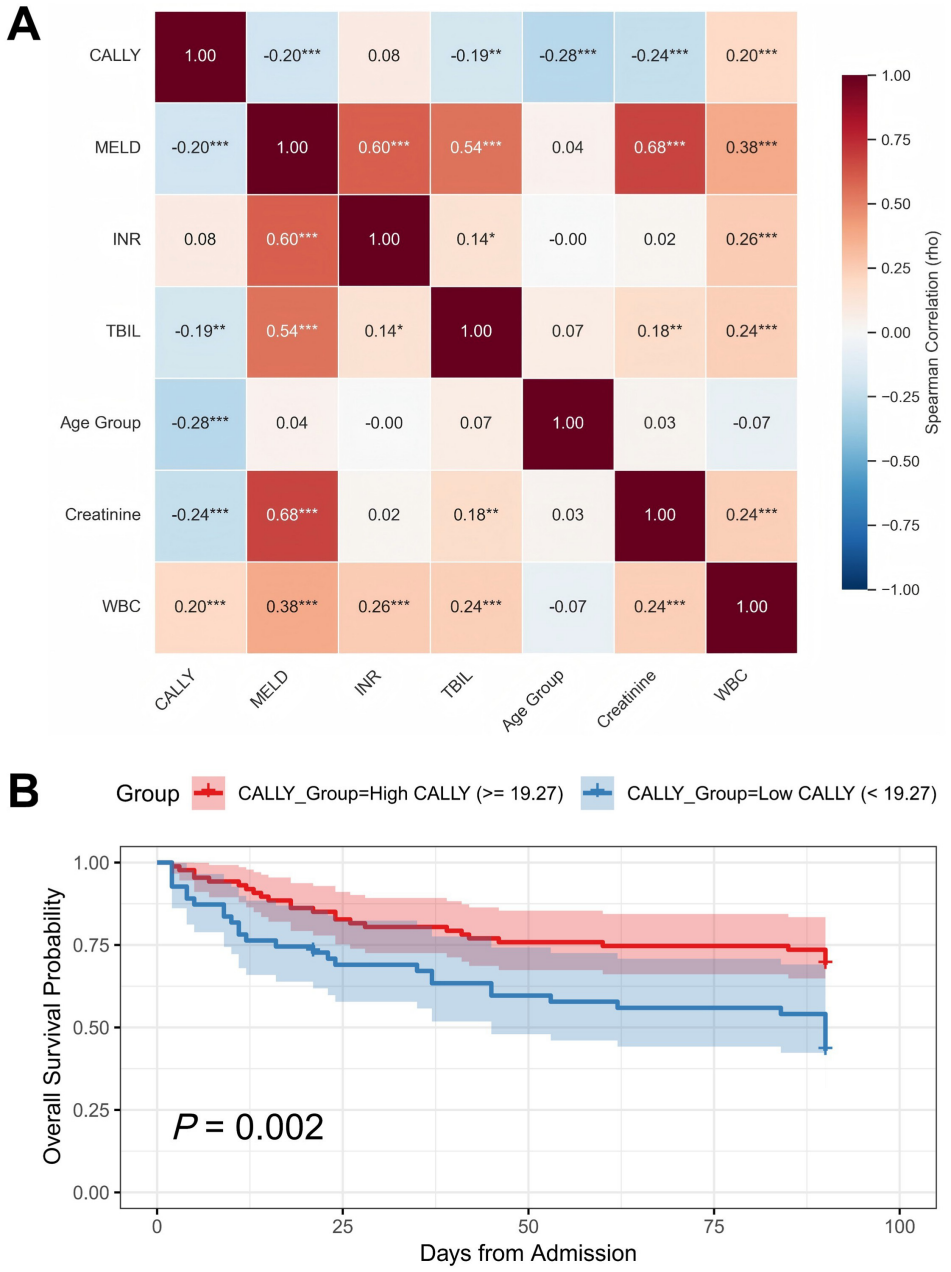
The Spearman correlation heatmaps and Kaplan-Meier survival curves of the training set. **(A)** The Spearman correlation heatmaps. The color gradient represents the Spearman correlation coefficient, where red indicates a positive correlation and blue indicates a negative correlation. The intensity of the color corresponds to the strength of the relationship. Asterisks denote statistical significance: * *P* < 0.05, ** *P* < 0.01, and *** *P* < 0.001. **(B)** Kaplan-Meier survival curves.

The baseline characteristics and laboratory data categorized by 90-day survival status are presented. Among the 329 participants in the training set, 195 were in the survival group and 134 were in the death group, resulting in a 90-day mortality rate of 40.7% (Table 1). Univariate analysis revealed that the CALLY Index, albumin, and lymphocytes were significantly higher in survivors than in non-survivors (Table 2). Conversely, the age, INR, TBIL, and MELD scores were significantly elevated in the death group (*P* < 0.05). Spearman correlation heat map shows that the CALLY index is significantly negatively correlated with MELD score (*r* = −0.20) and TBIL (*r* = −0.19; *P* < 0.05). Crucially, a lower CALLY value strongly indicates greater disease severity and a depleted physiological reserve (Figure 2a). It is worth noting that there is no significant linear correlation between the CALLY index and INR (*r* = 0.08, *P* > 0.05), which may reflect another dimension of physiological vulnerability, that is, immunonutrient reserve, which acts as an upstream driving factor independent of terminal coagulation failure (11). To ensure the robustness of the subsequent multivariate modeling, multicollinearity among the key predictors (CALLY index, log-TBIL, INR, age group, and creatinine) was rigorously assessed using the Variance Inflation Factor (VIF). All calculated VIF values were ranging from 1.098 to 1.339, indicating no significant multicollinearity and confirming the stability of the regression coefficients for the final 90-day mortality prediction model (Supplementary Table 2). Furthermore, survival curves demonstrated that patients in the high CALLY group (≥ 19.27) achieved a significantly superior survival rate compared to the low CALLY group (*P* = 0.002) (Figure 2b).

**Table 2:** Univariate analysis of potential predictors for 90-day mortality in the training set.

Variable	OR	95% CI	*P*
Total cholesterol	0.447	0.312–0.641	< 0.001
Triglycerides	0.443	0.273–0.718	0.001
HDL–C	0.132	0.038–0.454	0.001
LDL	0.494	0.325–0.752	0.001
Albumin	0.933	0.886–0.982	0.008
Prealbumin	0.99	0.980–1.001	0.073
AFP	0.997	0.995–0.999	0
Platelets	0.991	0.986–0.997	0.001
Total bilirubin	1.004	1.002–1.005	< 0.001
Direct bilirubin	1.005	1.003–1.008	< 0.001
Indirect bilirubin	1.001	0.999–1.003	0.444
ALT	1	0.999–1.000	0.129
AST	1	1.000–1.000	0.451
Total bile acid	0.999	0.997–1.001	0.276
Cholinesterase	0.999	0.996–1.002	0.583
GGT	0.999	0.998–1.001	0.389
INR	4.497	2.663–7.592	< 0.001
Prothrombin time	1.142	1.088–1.199	< 0.001
WBC	1.01	0.982–1.039	0.479
Hemoglobin	0.984	0.974–0.993	0.001
Monocytes	1.19	0.849–1.667	0.314
Neutrophils	1.051	0.996–1.109	0.07
Lymphocytes	0.345	0.204–0.583	< 0.001
CRP	1.066	1.030–1.103	0
Cystatin C	2.023	1.260–3.247	0.004
Creatinine	1.006	1.001–1.010	0.012
Urea	1	0.999–1.000	0.706
Sodium	0.899	0.854–0.947	< 0.001
Potassium	1.31	0.912–1.882	0.144
Phosphorus	0.951	0.470–1.927	0.89
Age Group	2.752	1.637–4.625	0
CALLY Index	0.983	0.972–0.993	0.002

### Feature selection and construction of the clinically parsimonious nomogram

**Figure 3:**
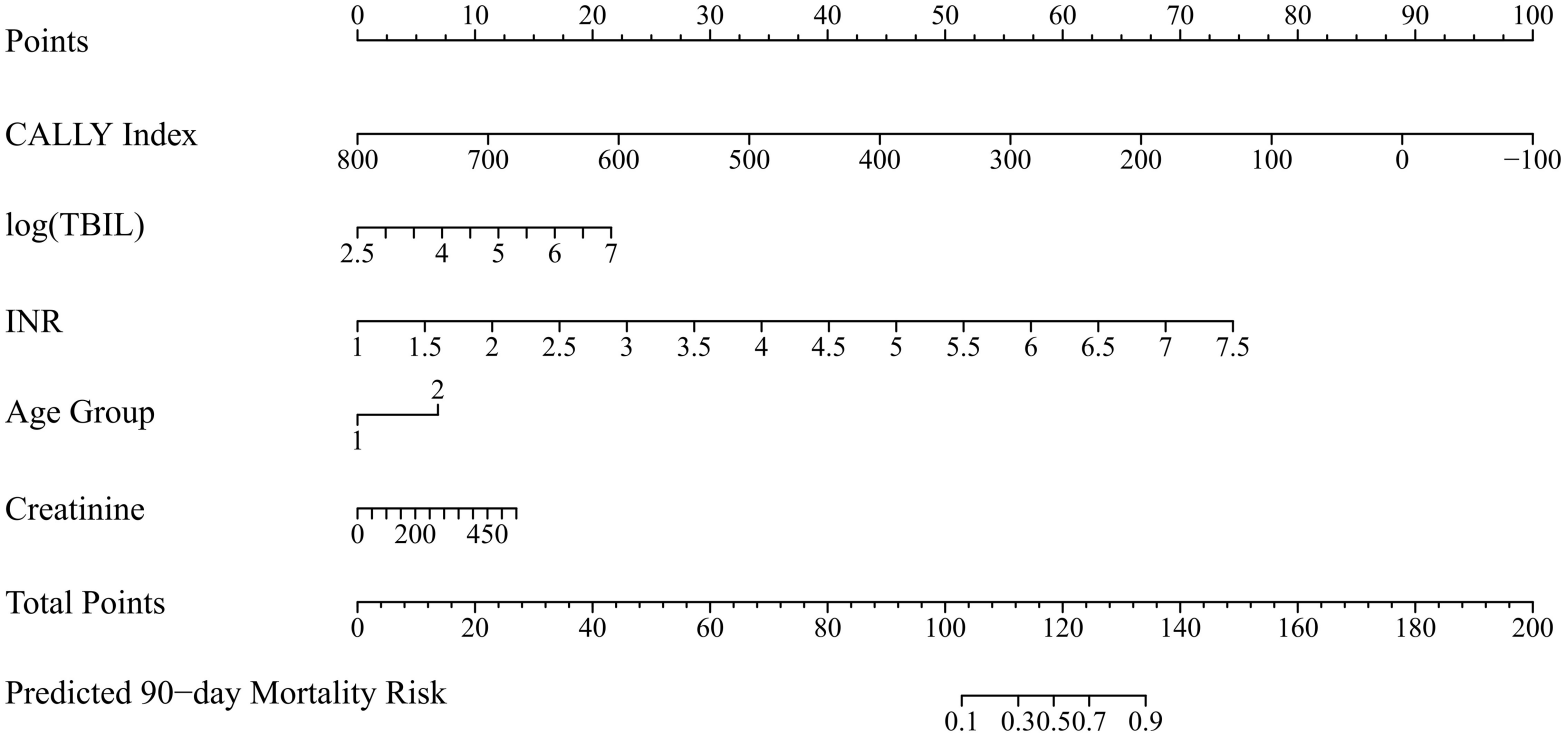
The nomogram for predicting 90-day mortality in patients with HBV-ACLF.

To streamline feature selection and reduce potential multicollinearity, we applied LASSO regression with 10-fold cross-validation. At the optimal lambda value, the CALLY index remained in the regularization path with a non-zero coefficient (−0.0239), suggesting a protective association (Supplementary Table 3). Next, variables identified by univariate analysis and LASSO were entered into a multivariable logistic regression model to evaluate independent prognostic value. In this analysis, the CALLY index remained an independent predictor of 90-day mortality (*P* = 0.012), together with log-transformed TBIL, INR, and age group (Table 3). Although creatinine did not reach statistical significance in this cohort (*P* = 0.143), we retained it in the final model because renal dysfunction is clinically important in ACLF. Finally, we constructed a bedside nomogram using these five parameters (CALLY index, log-TBIL, INR, age group, and creatinine) to support practical risk stratification (Figure 3).

**Table 3:** Multivariable logistic regression analysis of independent prognostic factors in the training set.

Variable	Coefficient	Std.Err	z	*P*	OR (95% CI)
CALLY Index	−0.0157	0.006	−2.511	0.012	0.984 (0.972–0.997)
log (TBIL)	1.5503	0.64	2.421	0.015	4.713 (1.343–16.527)
INR	1.6075	0.289	5.565	< 0.001	4.990 (2.832–8.793)
Age group	0.9633	0.298	3.229	0.001	2.620 (1.461–4.702)
Creatinine	0.0034	0.002	1.463	0.143	1.003 (0.999–1.008)
WBC	0.0043	0.013	0.32	0.749	1.004 (0.978–1.031)

### Incremental value of the immune-nutritional-inflammatory axis

**Figure 4:**
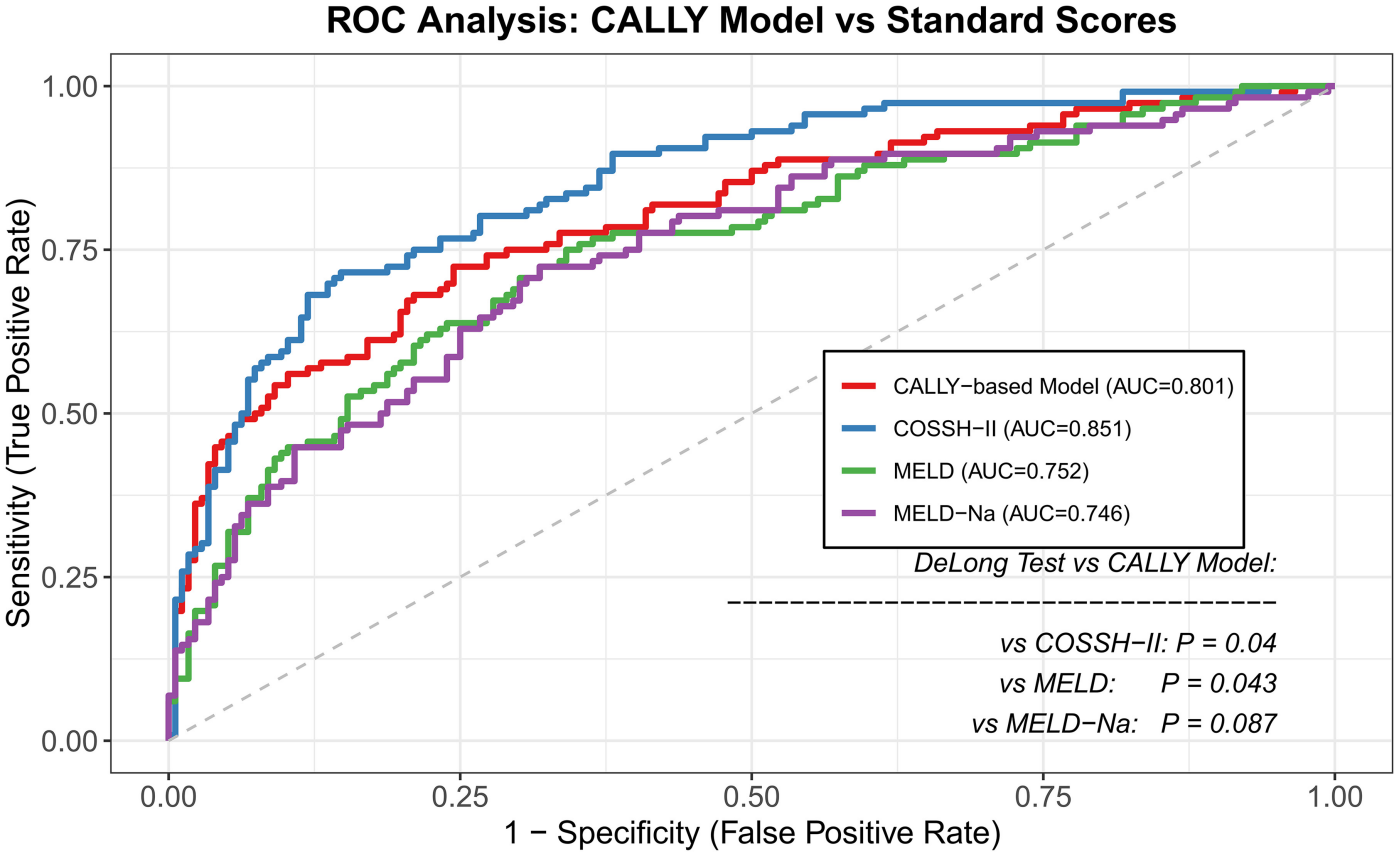
The DeLong test in the training set.

The clinical incremental value of CALLY was further quantified by a Continuous NRI of 0.6223 (*P* < 0.001) and an IDI of 0.0936 (*P* < 0.001), indicating improved risk reclassification and overall discrimination when immuno-nutritional status was incorporated (Table 4). The CALLY-based model (AUC 0.801) significantly outperformed the traditional MELD score (DeLong *P* = 0.043), demonstrating superior discriminative power for short-term mortality (Figure 4).

**Table 4:** The incremental value of CALLY.

Index	Estimate	95% CI	*P*
Continuous NRI	0.6223	0.3393–0.9601	< 0.001
IDI	0.0936	0.0377–0.2024	< 0.001

### Model consistency and performance of the CALLY-based nomogram

**Figure 5:**
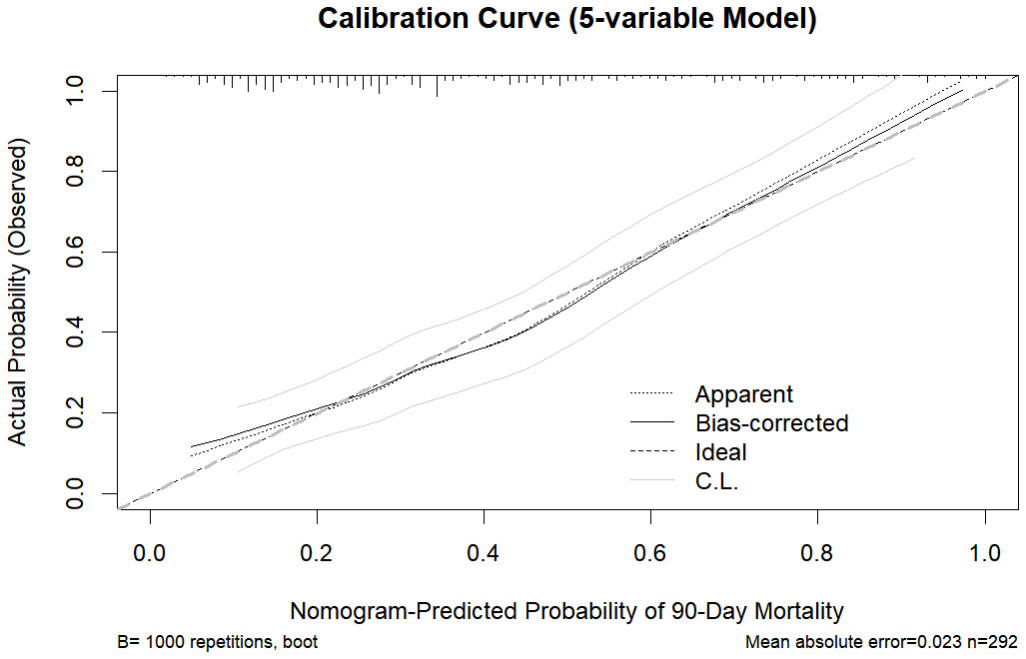
The calibration curve in the training set.

**Figure 6:**
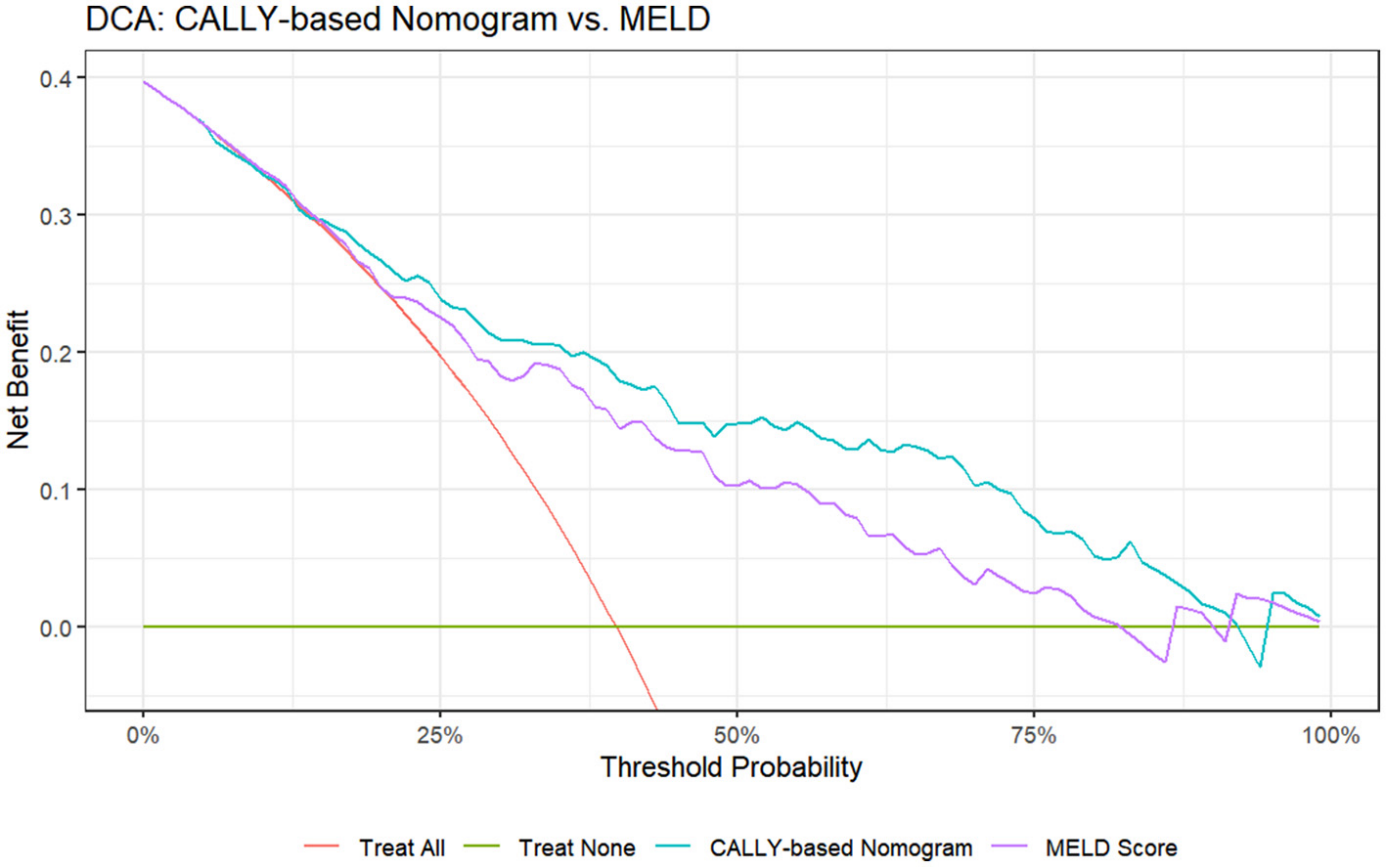
The decision curve analysis of the train set.

The calibration performance of the column diagram (Nomogram) based on CALLY is excellent. The deviation correction line in the calibration curve almost coincides with the ideal line (MAE = 0.023), indicating that there is no obvious over-fitting (Figure 5). DCA further confirmed the clinical utility of the model; the CALLY-based Nomogram provided a consistently higher net benefit than the MELD score across the majority of threshold probabilities (Figure 6).

### Machine learning performance and comparison

**Figure 7:**
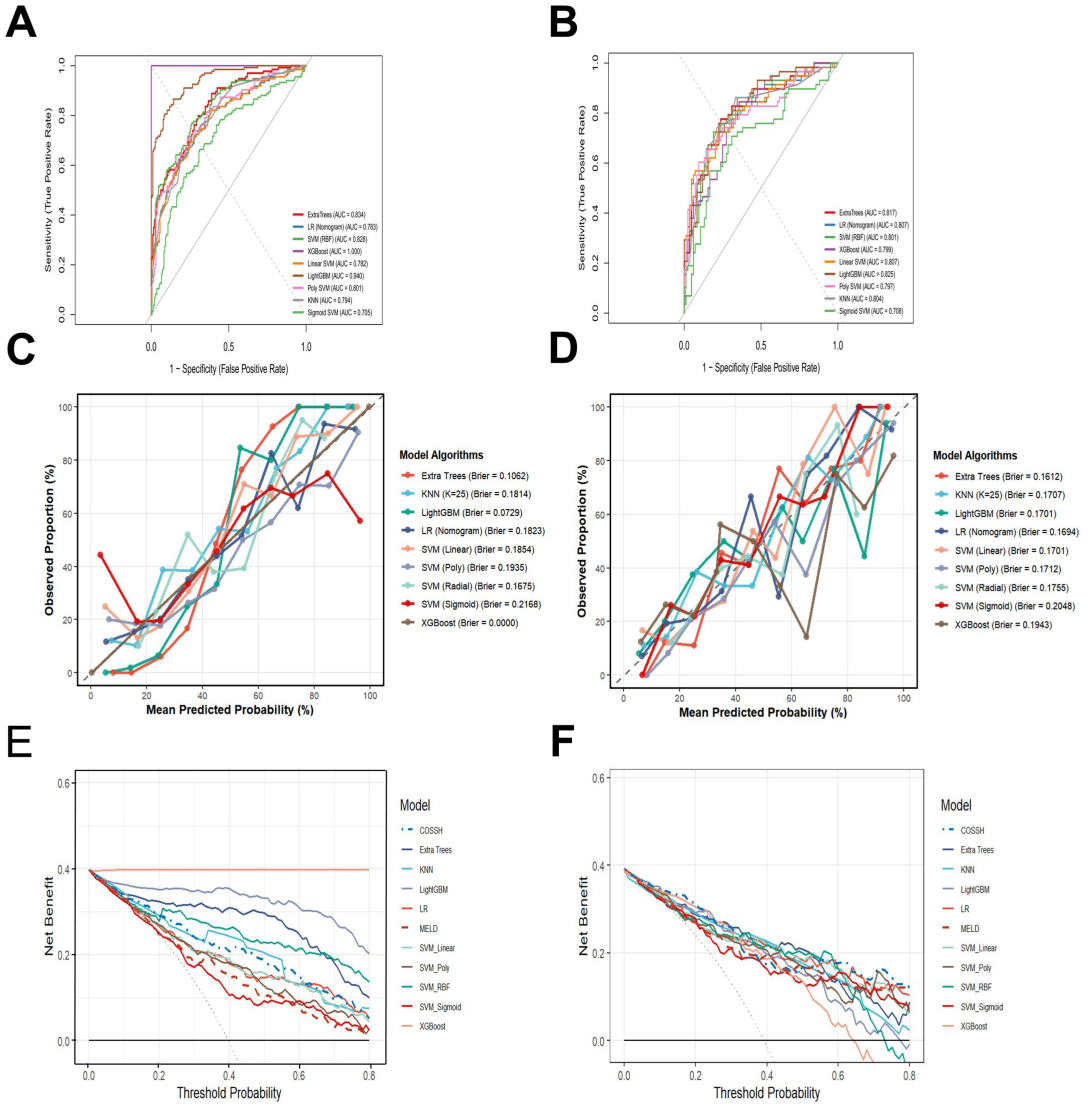
The AUC, Calibration curves, and DCA for the training and internal validation sets. **(A)** The AUC of the training set. **(B)** The AUC of the internal validation set. **(C)** The calibration curves of the training set. **(D)** The calibration curves of the internal validation set. **(E)** Decision curve for the training set. **(F)** Decision curve for the internal validation set.

Based on the CALLY Index and integrated clinical parameters (log-TBIL, INR, age group, and creatinine), the predictive efficacy of six machine learning algorithms was compared using 1,000 Bootstrap resamples (Table 5). Analysis of the experimental data showed that the LightGBM algorithm achieved the optimal overall performance, with an AUC of 0.940 (95% CI: 0.916–0.964) in the training set and 0.825 (95% CI: 0.757–0.894) in the internal validation set (Figure 7A, B). Furthermore, all evaluated algorithms demonstrated stable calibration properties (Figure 7C, D), and the decision curve analysis confirmed the superior clinical net benefit of the ML models compared to the baseline intervention strategies (Figure 7E, F).

**Table 5:** Machine learning modeling analysis in the training and internal validation sets.

Cohort	Model	AUC	AUC 95% CI	Acc	Acc 95% CI	Sen	Spe	PPV	NPV
Train	LightGBM	0.94	(0.916–0.964)	0.863	(0.821–0.898)	0.821	0.892	0.84	0.879
Test	LightGBM	0.825	(0.757–0.894)	0.775	(0.697–0.840)	0.672	0.845	0.75	0.789
Train	XGBoost	1	(1.000–1.000)	1	(0.989–1.000)	1	1	1	1
Test	XGBoost	0.799	(0.727–0.871)	0.725	(0.644–0.797)	0.845	0.643	0.62	0.857
Train	Extra Trees	0.834	(0.791–0.877)	0.76	(0.710–0.805)	0.5	0.938	0.848	0.732
Test	Extra Trees	0.817	(0.745–0.889)	0.768	(0.689–0.834)	0.776	0.762	0.692	0.831
Train	LR	0.783	(0.732–0.834)	0.729	(0.678–0.777)	0.552	0.851	0.718	0.735
Test	LR	0.807	(0.732–0.883)	0.761	(0.682–0.828)	0.724	0.786	0.7	0.805
Train	KNN	0.794	(0.745–0.843)	0.723	(0.672–0.771)	0.507	0.872	0.731	0.72
Test	KNN	0.804	(0.727–0.881)	0.746	(0.667–0.816)	0.862	0.667	0.641	0.875
Train	Linear SVM	0.782	(0.731–0.834)	0.726	(0.675–0.774)	0.47	0.903	0.768	0.713
Test	Linear SVM	0.807	(0.733–0.882)	0.761	(0.682–0.828)	0.724	0.786	0.7	0.805
Train	RBF SVM	0.828	(0.782–0.874)	0.76	(0.710–0.805)	0.597	0.872	0.762	0.759
Test	RBF SVM	0.801	(0.725–0.876)	0.775	(0.697–0.840)	0.724	0.81	0.724	0.81
Train	Poly SVM	0.801	(0.752–0.850)	0.733	(0.681–0.780)	0.515	0.882	0.75	0.726
Test	Poly SVM	0.797	(0.719–0.876)	0.782	(0.705–0.847)	0.603	0.905	0.814	0.768
Train	Sigmoid SVM	0.705	(0.646–0.763)	0.681	(0.627–0.731)	0.493	0.81	0.641	0.699
Test	Sigmoid SVM	0.708	(0.618–0.799)	0.725	(0.644–0.797)	0.569	0.833	0.702	0.737

### External validation and predictive superiority

**Figure 8:**
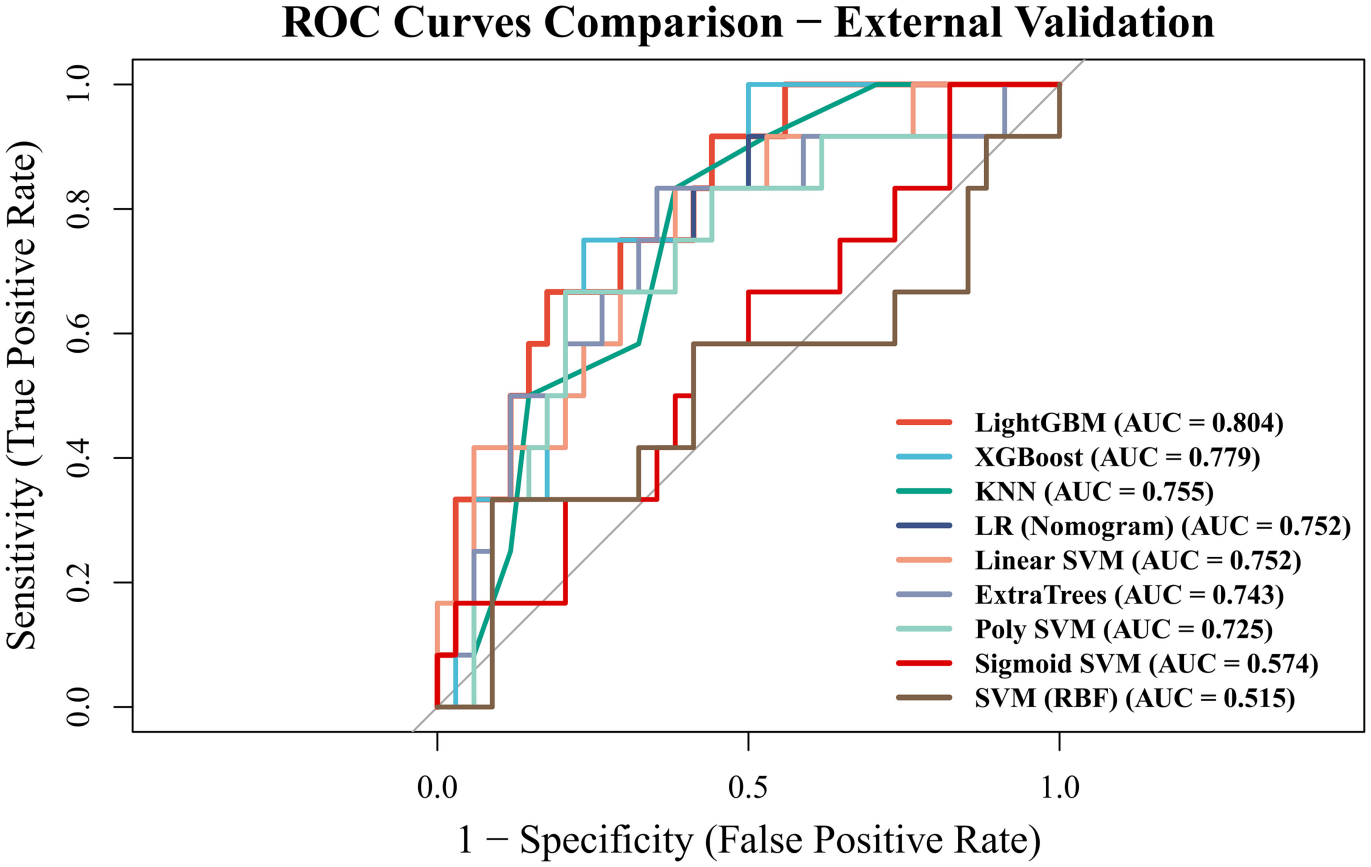
Test cohort for external validation of AUC.

**Figure 9:**
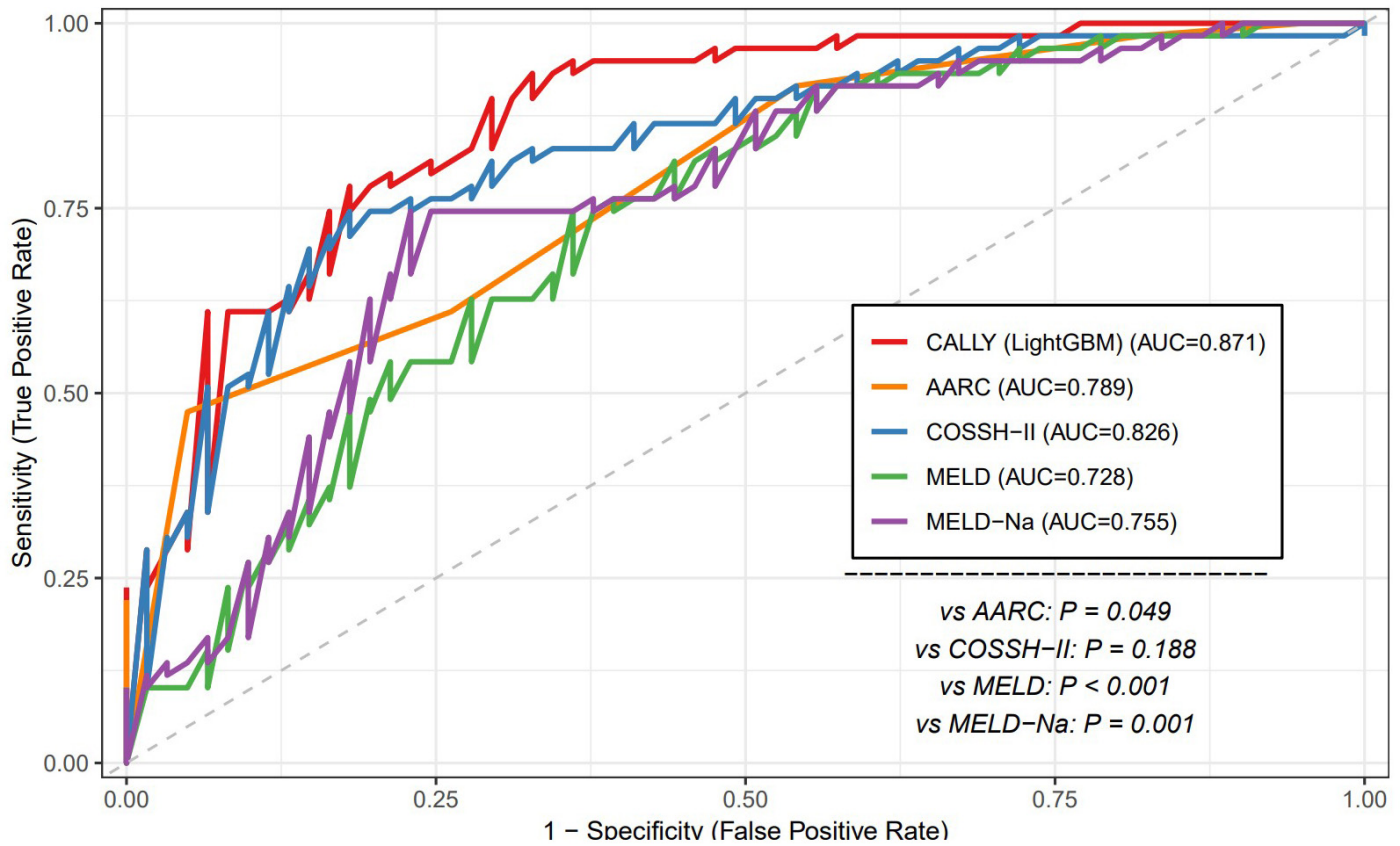
ROC curves of the CALLY model and AARC score.

To test general applicability, we performed temporal external validation (Figure 8). The LightGBM algorithm still performs best, with an AUC of 0.804, and shows stable distinguishing ability in independent patient cohorts compared with traditional scoring systems (Table 6). To further demonstrate the incremental value of our proposed framework, we conducted a head-to-head comparison with the AARC score in a validation subgroup of 138 patients with complete clinical profiles (Figure 9). Despite the restricted sample size, the CALLY LightGBM model exhibited a significantly superior discriminative capacity compared to the AARC score (AUC: 0.871 vs. 0.789; DeLong *P* = 0.049). This pivotal finding underscores that the synergistic integration of immune, nutritional, and inflammatory status provides a more precise and robust prognostic assessment than traditional scoring systems that rely heavily on time-sensitive or subjective parameters, such as blood lactate and hepatic encephalopathy (HE) grading.

**Table 6:** Machine learning modeling analysis external validation dataset.

Model	AUC (95% CI)	Acc (95% CI)	Sensitivity	Specificity	PPV	NPV
LightGBM	0.804 (0.669–0.939)	0.783 (0.636–0.891)	0.667	0.824	0.571	0.875
XGBoost	0.779 (0.641–0.918)	0.761 (0.612–0.874)	0.75	0.765	0.529	0.897
ExtraTrees	0.743 (0.571–0.915)	0.696 (0.542–0.823)	0.833	0.647	0.455	0.917
LR	0.752 (0.594–0.911)	0.652 (0.498–0.786)	0.833	0.588	0.417	0.909
KNN	0.755 (0.611–0.899)	0.674 (0.520–0.805)	0.833	0.618	0.435	0.913
Linear SVM	0.752 (0.594–0.911)	0.674 (0.520–0.805)	0.833	0.618	0.435	0.913
Poly SVM	0.725 (0.558–0.893)	0.761 (0.612–0.874)	0.667	0.794	0.533	0.871
Sigmoid SVM	0.574 (0.382–0.765)	0.391 (0.251–0.546)	1	0.176	0.3	1
RBF SVM	0.515 (0.295–0.734)	0.239 (0.126–0.388)	0.667	0.088	0.205	0.429

### Key predictors for HBV-ACLF risk prediction

**Figure 10:**
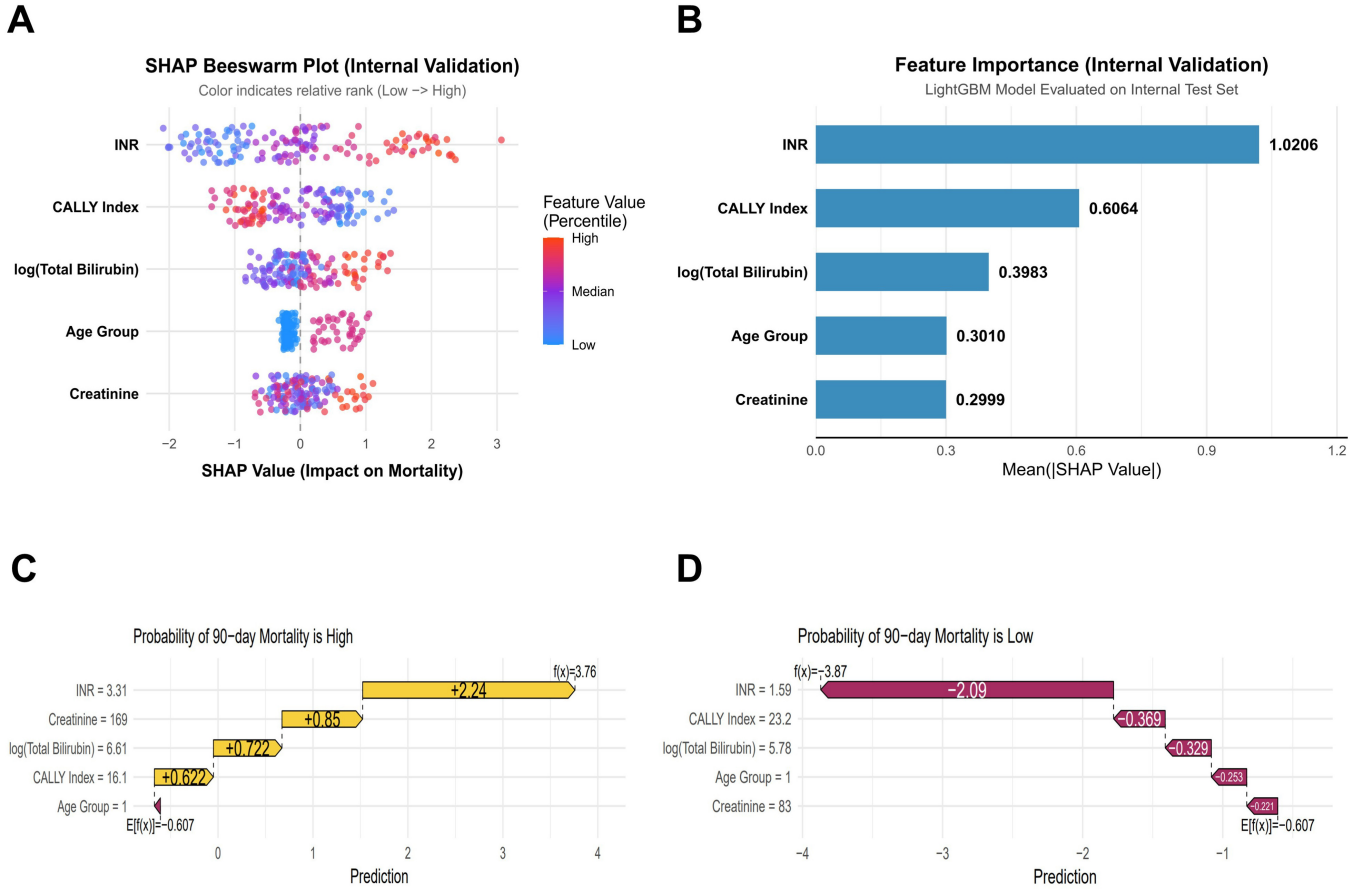
SHAP-driven explainability of an HBV-ACLF risk prediction model (Internal Validation). **(A)** SHAP BeesWarm. Each point represents an individual patient. The x-axis shows the SHAP value, indicating the direction and magnitude of each feature's contribution to the model output (positive values increase, negative values decrease the predicted outcome). Color encodes the feature value (red, higher; blue, lower). **(B)** SHAP Bar Chart. Ranks the predictors based on their mean absolute SHAP value, identifying INR and the CALLY index as the primary upstream drivers of patient survival. **(C)** A case of a patient with HBV-ACLF who died within 90 days (ID 2). Patient information: INR (3.31), Creatinine (169 μ mol/L), log (Total Bilirubin) (6.61), CALLY Index (16.10), and Age Group (1.00). **(D)** A case of a patient with HBV-ACLF who survived within 90 days (ID 132). Patient information: INR (1.59), CALLY Index (23.20), log (Total Bilirubin) (5.78), Age Group (1.00), and Creatinine (83 μ mol/L). This figure illustrates the SHAP values for two representative patients, showing the predicted log-odds of mortality and the specific contribution of clinical features. Yellow (right-pointing) arrows indicate a positive contribution to mortality risk, while purple (left-pointing) arrows indicate a protective effect.

We used the SHAP framework to further evaluate the interpretability of our model using the internal validation set at both global and individual levels. In the SHAP summary chart (Figure 10A), the distribution of each point on the x-axis reflects the intensity of the influence of each variable on the output of the entire queue model. The wider the distribution, the greater the contribution of the variable to predictive variability. In order to sort the predictors, we calculate the average absolute SHAP value (|SHAP|) of each feature and display it in the bar chart (Figure 10B). INR is identified as the most important predictor, followed by the CALLY index, logarithm-converted TBIL, age group and creatinine (Figure 10B). This sequence verifies the clinical paradigm, that is, although coagulation failure is the terminal event of ACLF, the potential immune-nutritional-inflammatory state (CALLY) is the main upstream driver affecting the survival rate. Representative individual explanations (ID 2 and ID 132) further illustrate how the model calculates risk for specific patients (Figure 10C, D). We present two typical cases to illustrate the model's logic: Case 1 (High Risk, ID 2): A patient predicted with a high 90-day mortality risk [f(x) = 3.76]. The plot illustrates how severe coagulation failure (high INR) and depleted immune-nutritional reserve (low CALLY index) synergistically e-nutritional-inflammatory state (CALLY) is ndpoint. Case 2 (Low Risk, ID 132): A survivor predicted with low risk [f(x) = −3.87]. In this case, the robust immune-nutritional reserve (high CALLY index) and stable organ functions acted as significant protective factors, shifting the prediction toward survival. These local explanations effectively bridge the gap between complex algorithmic outputs and bedside clinical decision-making, ensuring the model is both precise and interpretable. Together, these findings provide a robust and transparent basis for clinical risk stratification.

### Prediction website

To promote the accessibility and practical application of the model, we developed a dedicated website (https://4olggi-zz-zhang.shinyapps.io/hbv_aclf_app/). The platform is designed to assist physicians and researchers worldwide in utilizing our predictive model to estimate the 90-day survival probability for HBV-ACLF patients, thereby facilitating timely clinical decision-making and optimal ICU resource allocation.

## Discussion

The prognosis of HBV-ACLF is shaped by a complex interplay of liver injury, systemic inflammation, and immune dysfunction (12–15). Although systemic inflammation and immune disorders have been widely regarded as key drivers of organ failure and short-term death in acute liver failure (ACLF), the traditional MELD score mainly focuses on liver and kidney impairment (16). Therefore, this existing model ignores the key dimension of immune nutrition status, which is a key prognosis factor affecting survival (17–21). In this study, we propose to include the CALLY index in the risk stratification of hepatitis B virus-related acute-on-chronic liver failure (HBV-ACLF) as an important supplement to the traditional scoring system (22, 23). Our results further demonstrated that the CALLY-based LightGBM model significantly outperformed the AARC-ACLF score in a validation subgroup (AUC: 0.871 vs. 0.789; *P* = 0.049). This superiority likely stems from the fact that the CALLY index captures the immune-nutritional-inflammatory axis, which is the upstream physiological drivers, whereas traditional scores like AARC score rely heavily on end-stage events such as hepatic encephalopathy and lactate elevation. By identifying risks at this earlier, more intervention-amenable stage, our model provides a critical window for bedside decision-making. The biological significance of CALLY is that it can simultaneously characterize the multi-dimensional ability of inflammatory load (C-reactive protein), nutritional/synthetic state and vascular adaptability (albumin) and immune monitoring (lymphocytes). This comprehensive biomarker is effectively mapped into the LY index captures the immune-nutritional-inflammatory axis, which is the upstream physiologidyspensation. The existing literature emphasizes that the amplification of inflammation driven by PAMPs/DAMPs, coupled with the exhaustion of immune cells, will significantly increase the risk of secondary infection and multiple organ failure (24, 25). It is worth noting that as a negative acute protein, the decline in albumin reflects not only the intensity of the inflammatory response, but also the impairment of the body's physiological ability to antioxidant and remove endotoxins (26).

This approach may support earlier and more targeted triage in high-acuity settings. The predictive ability of the CALLY index is that it can reflect the overall response of the body to acute injury. As part of the CALLY framework, CRP can be used as an alternative indicator for systemic inflammatory response syndrome (SIRS), which is the main driver of multiple organ failure (MOF) (21, 27, 28). The lymphocyte count represents immune function, which decreases in the high-acuity settings. The predictive ability of thsecondary infection; while albumin reflects the failure of liver synthesis and exhaustion of systemic antioxidant capacity, which is crucial during sepsis (29–31). There is a significant negative correlation between the CALLY index and the MELD score (*p* < 0.05), which suggests that the CALLY index does reflect the decline of liver reserve function.

A key finding of this study is that integrating the CALLY index into the MELD framework can improve the accuracy of prognosis. Its continuous net weight classification index (NRI) is 0.6223, and the comprehensive discrimination improvement index (IDI) is 0.0936 (*P* < 0.001). Compared with the COSSH-II score, which focuses on advanced organ failure, the CALLY-MELD model provides a unique. Its continuous net weight classification index (NRI) is 0.acute injury. As part of the CALLY frnd our model uses easily available laboratory parameters (albumin, CRP, lymphocytes) to provide a convenient identification scheme to identify high-risk patients as soon as possible before multiple organ failure occurs (14). A notable methodological strength of our study is the inclusion of creatinine in the final LightGBM model, despite its univariate non-significance (*P* = 0.143). From a biological plausibility perspective, renal function is a well-established determinant of survival in HBV-ACLF; excluding it purely based on the *P*-value threshold could compromise the model's clinical integrity. Furthermore, within machine learning frameworks, feature selection prioritizes predictive gain and global contribution over individual *P*-values. Relying strictly on *P* < 0.05 for variable selection often risks over-fitting the model to the specific idiosyncrasies of the training cohort. By retaining this clinically gold-standard marker, we ensured that the CALLY-based model maintains robust generalizability in real-world ICU settings, where renal impairment frequently serves as a terminal prognostic event. Another critical observation in our study was the distinct trade-off between algorithmic complexity and generalizability. During the model development phase, the XGBoost algorithm achieved a perfect fit on the training data (AUC = 1.0) but showed a significant performance decline in the internal test set. This phenomenon suggests that for clinical datasets of modest size (*n* = 329), high-complexity ensemble methods may lean toward memorizing cohort-specific noise rather than identifying universal prognostic patterns.To ensure the model's clinical utility, we prioritized algorithmic robustness over extreme training-set performance. The LightGBM framework was ultimately selected as our proposed model because it maintained a superior and stable performance gradient (AUC of 0.940 in training vs. 0.825 in internal testing). This stability indicates that LightGBM effectively captured the non-linear interactions within the immune-nutritional-inflammatory axis while resisting the risk of overfitting. These findings underscore that in high-acuity conditions like HBV-ACLF, the generalizability validated by temporal cohorts is far more valuable for bedside decision-making than a perfect fit on retrospective derivation data. In this study, the LightGBM model demonstrated superior performance over traditional Logistic Regression (LR) and static scoring systems. While LR is a cornerstone of clinical modeling, it is inherently limited by the assumption of linearity between variables. In contrast, the pathophysiology of HBV-ACLF is a complex, multi-organ syndrome driven by an intense cytokine storm and fluctuating nutritional status. Machine learning algorithms like LightGBM excel at identifying non-linear interactions within the immune-nutritional-inflammatory axis, while interactions that linear models often fail to capture. In addition, while several high-performance models published between 2021 and 2026 have leveraged advanced radiomics or multi-omics technologies to achieve exceptional accuracy (AUC up to 0.98), their integration into emergency ICU workflows remains challenging due to high costs and long turnaround times (Supplement Table 3) (12, 14, 32, 33). In contrast, our CALLY-based model achieves a robust and stable prognostic performance (AUC 0.804–0.825) using only zero-latency, routinely available markers. This strategic focus on clinical parsimony ensures that our model can provide life-saving risk stratification within the first hour of admission, representing a significant practical optimization over complex research tools. Furthermore, the incorporation of SHAP addresses the black-box nature of AI. By providing individualized feature attribution, clinicians can clearly perceive how specific markers shift a patient's risk profile. SHAP analysis is used to explain these interactions, and the results show that the INR and CALLY indexes are the most influential predictors. SHAP analysis was used to explain these interactions, and the results show that the INR and CALLY index are the most influential predictors. To ensure the robustness of these findings, we further performed a validation using LightGBM's native importance metrics (Information Gain, Cover, and Frequency). This dual-validation approach consistently identified the INR and CALLY index as the core prognostic drivers, with the CALLY index exhibiting the highest contribution in the Gain-based evaluation (Supplementary Figure 1). Such methodological convergence reinforces that our identified predictors are not merely statistical artifacts but are grounded in the complex immunopathology of HBV-ACLF. This aligns with the clinical understanding that while coagulation failure is the terminal event in ACLF, the immune-nutritional reserve (CALLY) plays a critical role in survival. This level of transparency, combined with the model's stable performance across a contemporary validation cohort (2024–2025), confirms that our framework is not only statistically robust but also clinically actionable in modern intensive care settings. While the cally index threshold of 19.27 demonstrated significant prognostic value in our study, its clinical utility as a definitive risk stratification red line warrants further validation through multi-center prospective cohorts. Patients below this threshold are ultra-high-risk groups and need early intervention, including nutritional support and monitoring of secondary infection as soon as possible. In addition to statistical significance, DCA analysis also shows that the CALLY-based model continues to show better net clinical benefits within a wide range of threshold probability, and its clinical practicality is higher than the traditional MELD score. This is crucial in practical clinical practice, because early intervention, such as liver transplant evaluation or activation of artificial liver support system (ALSS), is crucial. The column diagram constructed in this study provides a convenient bedside decision support tool to simplify the output of complex models into actionable clinical decision-making, so as to realize individualized medical care. The superiority of our model does not merely stem from the algorithmic prowess of ML, but from the synergistic alignment between the CALLY index and the immunopathology of HBV-ACLF. Unlike sepsis-related liver failure where infection is the primary driver, HBV-ACLF involves a profound depletion of nutritional reserve (Albumin) and immune exhaustion (Lymphocytes) coupled with intense sterile inflammation (CRP). By focusing on this triad of the immune-nutritional-inflammatory axis, our framework captures the unique biological signature of HBV-driven liver injury more effectively than traditional scores. This study, therefore, represents a shift from general risk assessment toward phenotype-optimized precision prognosis.

Although our research results are encouraging, some limitations still need to be considered. As a retrospective study, inherent choice bias is a matter of concern, although the MICE method helps to mitigate the impact of data loss. A potential limitation of our approach is the reliance on a single baseline assessment. HBV-ACLF is undeniably a dynamic process where inflammatory and nutritional markers fluctuate. However, our model focuses on the Admission Phenotype. Given that Albumin, Lymphocytes, and CRP are part of the early systemic inflammatory response syndrome (SIRS), their baseline levels capture the intensity of the initial immunological hit.Furthermore, in a real-world retrospective setting, the availability of laboratory data at fixed intervals (e.g., precisely on Day 3 or Day 7) is often inconsistent, which would introduce significant selection bias if a longitudinal model were applied to the entire cohort. By demonstrating that a minimalist, admission-based LightGBM model can maintain an external AUC of 0.804, we provide a tool that is both methodologically stable and clinically actionable during the most critical early phase of ICU care. And we have formally identified dynamic CALLY modeling as a primary objective for our upcoming prospective multi-center study. In addition, this was a retrospective, single-center study, which inherently carries risks of selection bias and may limit the immediate generalizability of our findings to other geographic regions. To mitigate this, we performed a temporal validation using a contemporary cohort (2024–2025) and achieved a robust AUC of 0.804. However, we recognize that multi-center validation is essential for further confirming the model's stability. Consequently, we are currently in the early stages of establishing a multi-center collaborative network with other regional tertiary hospitals to initiate a prospective validation study. This will specifically focus on the real-time performance of the CALLY index across different HBV genotypes and regional treatment variations, ultimately refining the model for broader clinical application.

## Conclusion

In conclusion, the CALLY index provides a clinically interpretable approach for predicting 90-day mortality in patients with HBV-ACLF. By explicitly incorporating the immune–nutritional–inflammatory axis, this model improves upon conventional scoring and may help clinicians with early triage, ICU resource allocation, and more personalized critical care. Further prospective studies are needed to refine and validate this approach, paving the way for precision medicine in HBV-ACLF management.

## Data Availability

The raw data supporting the conclusions of this article will be made available by the authors, without undue reservation.
