# Supplementary material for: A machine learning model for 90-day mortality prediction in hepatitis B virus-related acute-on-chronic liver failure: the pivotal role of CALLY index
Source: Front Med (Lausanne). 2026 Jul 3;13:1814799. doi: 10.3389/fmed.2026.1814799 (PMC13375720; doi:10.3389/fmed.2026.1814799)
Supplement: Supplementary file 4 [file Data_Sheet_1.PDF]

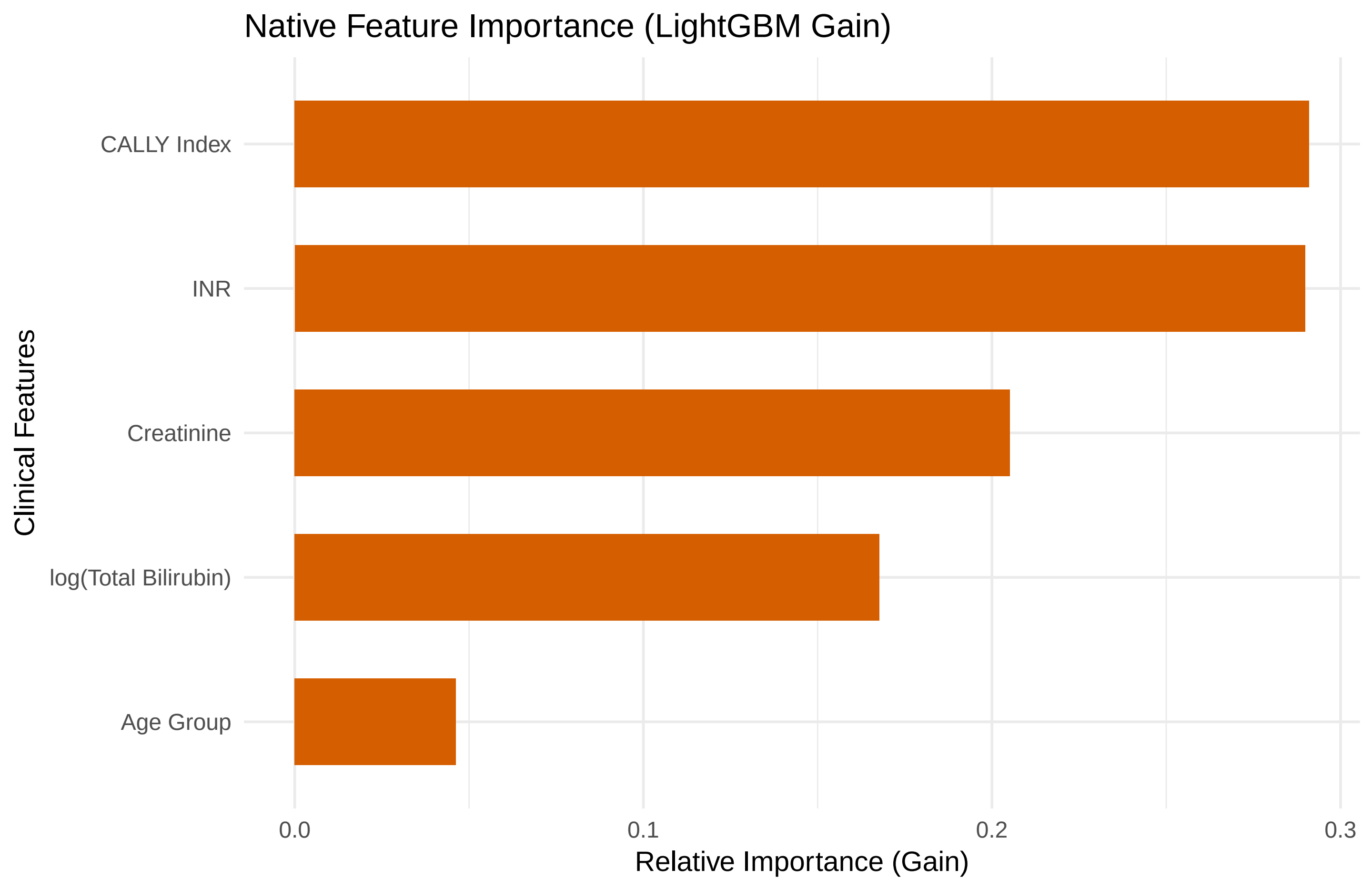

Native Feature Importance (LightGBM Gain)
Clinical Features
CALLY Index
INR
Creatinine
log(Total Bilirubin)
Age Group
0.0
0.1
0.2
0.3
Relative Importance (Gain)
